# A study protocol for a cluster randomized controlled trial to test the applicability of the South African diabetes prevention program in the Eastern Cape Province of South Africa

**DOI:** 10.1186/s12889-022-14884-1

**Published:** 2023-01-31

**Authors:** Jillian Hill, Yandiswa Yako, Cindy George, Hannibal Musarurwa, Esme Jordaan, Andre P. Kengne

**Affiliations:** 1 Non-Communicable Diseases Research Unit, South African Medical Research Council, Cape Town, South Africa; 2 Department Human Biology, Faulty of Health Sciences, Walter Sisulu University, Mthatha, South Africa; 3 Biostatistics Unit, South African Medical Research Council, Cape Town, South Africa

**Keywords:** Diabetes prevention, Community-based intervention, Behavioural change, Lifestyle intervention, Translational research, South Africa

## Abstract

**Background:**

Convincing evidence supports the effectiveness of lifestyle interventions in preventing the occurrence of diabetes in high-income countries, however little is known about appropriate interventions for use in African countries, where there are higher relative increases in diabetes prevalence. The South African Diabetes Prevention Programme (SA-DPP) was initiated with the aim of preventing or delaying the occurrence of diabetes among South Africans (SAs), through interventions, targeting lifestyle changes related to diet and physical activity. The purpose of the current project is to implement and evaluate the suitability and applicability of the SA-DPP developed and tailored in urban populations in the Western Cape Province, in peri-urban populations in the Eastern Cape Province of SA.

**Methods:**

The SA-DPP, which is an cluster randomized control trial, will be implemented in adults aged 30–65 years residing in the OR Tambo district, Eastern Cape, SA. Participants will be recruited using self-selected sampling techniques and 24 clusters across peri-urban communities will be randomly allocated to participate in the lifestyle intervention, facilitated by non-professional health workers (NPHW). The diabetes risk screening will follow a two-staged approach, including the community-based screening, using the African diabetes risk score (ADRS), followed by a clinic-based risk status assessment by an oral glucose tolerance test (OGTT) to exclude unknown diabetes. The lifestyle-change objectives of the current programme relate to, 1) < 30% of total energy intake from fat; 2) < 10% of total energy intake from saturated fat; 3) > 15 g of fibre/1000 kcal; 4) > 4 h/week moderate level of physical activity; and 5) > 2% body mass index (BMI) reduction.

**Discussion:**

The SA-DPP could represent a successful model for the prevention of diabetes and potentially other lifestyle-related diseases in SA and other countries in the region that are confronted with similar challenges.

**Trial registration:**

PACTR202205591282906.

**Supplementary Information:**

The online version contains supplementary material available at 10.1186/s12889-022-14884-1.

## Background

The global prevalence of diabetes mellitus has substantially increased over the past few decades [1]. According to the latest global estimates, 537 million (10%) of adults are currently living with diabetes, with Africa predicted to experience the largest relative increase of new cases over the next 24 years [1]. Indeed, it is projected that the population with diabetes in Africa, which is mainly driven by type 2 diabetes mellitus (T2DM) [2], will increase by 129% from 24 million to 55 million by 2045 [1]. South Africa has the highest age-adjusted prevalence of diabetes in adults aged 20–79 years in the African region [2], with the greatest burden experienced in socio-economically disadvantaged communities. It is further estimated that at least a similar proportion of adult South Africans without diabetes, are at high risk of developing the disease in future.

Community-based risk screening and prevention strategies, like lifestyle interventions, are frequently advocated to reduce the growing global burden of T2DM. However, despite the convincing evidence from high-income countries on the effectiveness of lifestyle interventions in preventing diabetes among high-risk individuals [3, 4], little is known about implementing these interventions in real-life settings in Africa. The South African Diabetes Prevention Programme (SA-DPP) was initiated by the Non-Communicable Diseases Research Unit (NCDRU) of the South African MRC (SAMRC) to generate such evidence for South Africa (SA) that could inform similar initiatives in other countries in the African region. The SA-DPP is an intervention, which was developed and tailored in urban populations, comprising three components, namely, [1] home/community-based screening by non-professional health workers (NPHW) to detect those at high risk of developing T2DM using a non-laboratory-based diabetes risk score (the African Diabetes Risk Score, ADRS) [2, 5]; delivery by NPHW of group-based task-oriented and culturally tailored socio-behavioural counselling targeting the lifestyle change objectives achieved in the Finnish Diabetes Prevention Study (DPS) [i.e. A) < 30% of total energy intake from fat; B) < 10% of total energy intake from saturated fat; C) > 15 g of fibre/1000 kcal; D) > 4 h/week moderate intensity physical activity; and E) > 5% weight reduction [3, 6]; structured cell-phone messaging to enhance program adherence and retention. While the SA-DPP is adapted from programmes previously shown to be effective in Finland [6], Australia [7], and India [8], it has undergone extensive adaptation to be culturally appropriate for South Africa (see Additional file 1 for intervention descriptions). For the SA-DPP to be relevant to the greater South African population it is important to test the intervention in other South African communities. The OR Tambo District Municipality in the Eastern Cape Province, South Africa, was selected because of the continuously increasing proportion of newly diagnosed T2DM cases observed [9].

The current protocol is for a project to implement and evaluate in peri-urban populations in the Eastern Cape Province, the suitability, applicability, appropriateness, and success of the SA-DPP developed and tailored in urban populations in the Western Cape Province of South Africa.

## Methods

### Study objectives

The primary objective of this study is to determine the weight change of participants receiving the lifestyle intervention at the end of a 12-month period. We are testing the hypothesis that the SA-DPP lifestyle intervention can prevent or delay the occurrence of diabetes among high-risk South Africans, by demonstrating that T2DM incidence during follow-up is lower among those who receive the intervention compared to those in the control group. The resources required to demonstrate such an effect, which include large sample sizes, long duration of follow-up and costly biochemical investigations, are far beyond what can be achieved in most settings. However, literature shows that change in body adiposity, which is apparent within few weeks-to-months, is a very strong predictor of future change in diabetes incidence. Accordingly, the outcome measure for this objective is to evaluate change in weight and consequent body mass index (BMI).

Our secondary objectives are to determine, 1) the level of attainment of lifestyle intervention objectives (including dietary, physical activity); 2) mean differences in fasting blood glucose, HbA1c, BMI; 3) changes in blood pressure, lipid profile and waist circumference; 4) change in insulin sensitivity; 5) change in diabetes risk score and 6) incident diabetes.

Our tertiary objectives include, 1) descriptive analysis of the process involved in the intervention; 2) retrieving qualitative feedback from intervention participants; 3) conducting analysis of impact on lifestyle (i.e., quality of life, dietary and physical activity behavioural changes) and 4) evaluating the economic impact of the intervention.

### Study design

This study is a fixed matched unblinded randomized cohort design with 12 clusters per arm. To reduce between-unit (between-cluster) variability in the estimation of the intervention effect, reduce the standard error and increasing power and precision [8, 10], clusters will be organized into pairs, with one cluster within each pair assigned to a study arm. Twenty-four (24) clusters (12 intervention and 12 control) across peri-urban communities in OR Tambo district in the Eastern Cape, South Africa will be randomly allocated to participate in a lifestyle intervention facilitated by teams of NPHW.

### Site selection and sampling frame

The OR Tambo District Municipality is located on the coastline at the east end of the Eastern Cape Province, South Africa. The district consists of five local municipalities, namely King Sabata Dalindyebo, Nyandeni, Mhlontlo, Port St Johns and Ingquza Hill and it is one of the four Integrated Sustainable Rural Development Programme (ISRDP) nodes in the Eastern Cape Province. The district has a population of 1,457,384 with a population growth of 1.47% annually. According to the most recent statistics, 99.04% of the population is black, with 25.35% (*n* = 345,944) between the age of 30 and 65 years.

### Study population

The study population will comprise South Africans aged 30–65 years, permanently residing in the OR Tambo district. The justification for the age bracket is motivated by, 1) the fact that available tools for T2DM risk screening at the community level have been designed and validated for use in this age group; 2) most people at risk of T2DM are likely to be found in this group; and 3) intervention in a more elderly population may require programmes that are specific to that age range [11]. Our pilot project in Cape Town, South Africa, has also indicated that among people at high risk of diabetes, based on a risk score, and further go on the be at high risk, by oral glucose tolerance test, are typically aged older than 30 years [12]. Further criteria for inclusion into our study includes being fluent in Xhosa or other dominant languages in the area (Afrikaans, English), being able to give informed consent and willing to participate in a lifestyle intervention trial. We will exclude individuals with known or screen-detected diabetes, active tuberculosis, major illness, or those that are bedridden. Individuals with severe mental illness or substance abuse will also be excluded as these conditions may likely interfere with participation and/or ability to consent. Finally, we will exclude individuals using medication that can affect glucose tolerance and women that are currently pregnant or planning for a pregnancy.

### Sample size calculation

To detect a minimum clinically relevant mean weight difference of 2 kg (±5.8, standard deviation) between intervention and control clusters [13], assuming an 80% power, a two-sided alpha of 5% and an intra-cluster correlation coefficient (ICC) of 0.02 [14], and accounting for a 20% drop-out during follow-up, we require 12 clusters of 20 participants per study arm; equalling 24 clusters and 480 participants. Using the same assumptions as above, Table 1 below presents the total number of clusters and participants required for various cluster sizes. For a population with an average starting weight of 80 kg, a mean weight difference of 2 kg correspond to 2.5% change, with the study being adequately powered to detect even larger effect sizes, such as those achieved in the Finnish DPS. A 1 kg change in weight at 1 year is associated with approximately 16% reduction in T2DM incidence at 3 years [15]. The sample size was calculated using the CRTSize package version 1.0 (R statistical software; The R Foundation for Statistical Computing Platform).

**Table 1:** Total number of clusters and participants required for various cluster sizes

Number per cluster	5	6	8	10	12	15	16	18	20
**Number per cluster accounting for 20% drop-out**	7	8	10	13	15	19	20	23	25
**Total clusters**	60	52	40	34	30	26	**24**	22	22
**Total sample**	420	416	400	442	450	494	**480**	506	550

### Recruitment processes

**Fig. 1:**
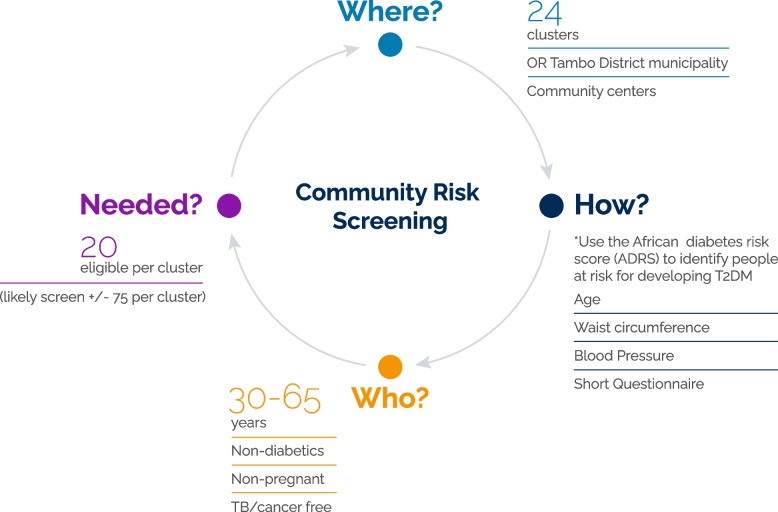
Community screening

Community-based screening (Fig. 1) will be conducted in the O.R. Tambo district (Additional file 2: Map of OR Tambo district). The district consists of nine cities, namely: Mthatha (previously Umtata), Tsolo, Port St Johns, Qumbu, Flagstaff, Libode, Lusikisiki, Mqanduli, Ngqeleni (Additional file 2: Map of OR Tambo). Six clusters (suburbs/townships) will be selected from the largest town (Mthatha), that has a population size of approximately 96,111 people. Then, four clusters from Tsolo (7794 people) and two clusters each will be selected from the seven smaller towns, where population density varies between approximately 2629 to 6500 people. Following community entry and buy-in, information flyers will be distributed to community members inviting them to attend various screening sites at a central community venue (clinic/community centre). Eligible community members deemed at high risk of developing diabetes, using the African Diabetes Risk Score (ADRS) [5] will be invited for further baseline evaluation and to participate in the intervention study. The ADRS [5] is based on three variables (age, waist circumference and hypertension status), and has been shown to have a good predictive accuracy (c-statistic = 0.82 [95%CI: 076–0.87]) during external validation in Black South Africans [5]. Baseline assessments will occur at the nearest clinic or community health care facility or in a mobile clinic if the area is too remote.

Twenty-five participants per cluster will be recruited (oversampling to account for 13–20% expected to have undiagnosed diabetes) to undergo the full baseline evaluation, including oral glucose tolerance tests (OGTTs) and glycated haemoglobin (HbA1c). Participants classified as having diabetes at this point will be excluded from the study and suitably referred.

Consenting eligible participants that fulfil the inclusion criteria will be required to complete a brief questionnaire and examination to complete the diabetes risk evaluation. Those identified as high risk via the ADRS will be assigned to commence with the intervention at the randomly pre-defined time-point for their cluster. Clusters will be created according to residential areas of participants.

### Randomisation

Matched pairing will be done for clusters according to the information available for the population, including for example, size of the cluster, whether they are peri-urban or rural and for factors that are likely to be associated to the outcome. Members of the paired clusters will then be randomized to an intervention or control arm. The differences between the paired clusters after the intervention are then most likely due to the intervention.

### The intervention

The SA-DPP is an intervention comprising three components, namely, [1] home/community-based screening by non-professional health workers (NPHW) to detect those at high risk of developing T2DM using a non-laboratory-based diabetes risk score (ADRS) [5] [2]; delivery by NPHW of group-based task-oriented and culturally tailored socio-behavioural counselling targeting the lifestyle change objectives achieved in the Finnish Diabetes Prevention Study (DPS) [i.e. A) < 30% of total energy intake from fat; B) < 10% of total energy intake from saturated fat; C) > 15 g of fibre/1000 kcal; D) > 4 h/week moderate intensity physical activity; and E) > 5% weight reduction [6] [3]; structured cell-phone messaging to enhance program adherence and retention. NPHW will undergo 2-weeks of training using a standardized programme, training manuals, and practical exercises.

#### Delivery of the intervention (adapted from the SA-DPP to suit funding timeline)

The intervention will be delivered in six sessions (excluding one introductory session) of 2 hours each, over a seven-week period, with SMS text messages to support the intervention from programme initiation for a period of 9 months. The six group sessions will include education and goal setting tailored around, diabetes and its prevention, diet and nutrition, physical activity, smoking, alcohol, and stress. The overall intervention is based on empowerment ideology [16], emphasising the participant’s ability to make informed choices, and his/her role as an independent decision-maker taking responsibility and regulates his/her own actions. The role of NPHW (assisted by nurses and/or dieticians) is to facilitate and moderate the discussion, giving assignments and strengthening the peer support role of the groups. Text-messaging will be used to facilitate the group session, engage participants in discussions, and for ongoing support and motivation after the group intervention. Text messages will be sent twice weekly. It will be informed by the model of the Indian text-messaging diabetes prevention trial [17]. The programme will also encourage accessing to local community resources and support in the intervention cluster, as is recommended and extensively used in diabetes prevention programmes (DPP) elsewhere [18].

The SA-DPP work package tools have been developed and validated in the SA-DPP population and contains a curriculum booklet, a participant workbook, and a facilitator workbook. The curriculum booklet and participant workbook has been translated into isiXhosa, which in the most spoken language in the Eastern Cape.

#### Duration of intervention and follow-up evaluations

The total duration of face-to-face intervention is 7 weeks and the planned duration of follow-up from baseline to 12 months. Participants will receive twice weekly maintenance support in the form of text-messaging for a period of 9 months from the start of the intervention.

#### Control groups

Following screening, participants in the control arm of the study will receive standard care. As there is no uniform management in South Africa for those at high risk of developing T2DM, the participants in the control arm will receive advice in the form of a healthy living resource guide that has been used in similar trials [19]. This guide will comprise culturally appropriate written and pictorial information about healthy lifestyle and lowering diabetes risk. At the end of the study, the intervention will be offered to all participants.

### Data collection

#### Diabetes risk screening

Diabetes risk screening will follow a two-staged approach: a community-based risk screening using a risk questionnaire, followed by a clinic-based risk status assessment using biochemical analyses.

#### Community-based diabetes risk screening

**Fig. 2:**
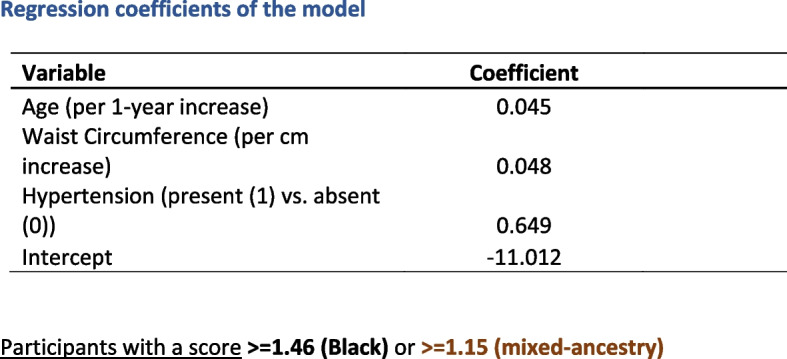
The African diabetes risk score coefficients

Trained fieldworkers will conduct the community-based risk screening in community centres by administering a brief screening questionnaire (age, gender and ethnicity) and measure anthropometry and blood pressure (BP). Three BP measurements will be taken at two-minute intervals using an Omron BP monitor (COMFORT M6), after the participant had been seated for 5 minutes. Height, weight, and waist measurements will be measured using standardized techniques. These measures will be used to estimate the risk of T2DM by the ADRS [5] (Fig. 2). Participants deemed at high-risk will be referred to our research clinic for biochemical investigations. Participants with blood pressure levels considered to constitute an immediate risk for their health (SBP ≥ 140 and or DBP ≥ 90 mmHg) will be referred to a nearby public health facility for further management.

#### Clinic-based diabetes risk confirmation and baseline measurements and follow-up measurements after 12 months

Participants deemed at high-risk during community-based screening will be transported to our research clinic for further assessment. Baseline assessments includes an OGTT (to exclude participants with previously undiagnosed diabetes, who will be referred for treatment) and other biochemical and clinical assessments. Blood samples for glucose and lipids will be drawn after a 10 hour overnight fast, followed by a standard OGTT, using 75 g of anhydrous glucose in 250 ml of water will be administered, and blood samples taken 120 minutes later [20]. A qualified nurse will collect the blood samples. All biochemical analysis will be conducted by locally accredited laboratory in Mthatha according to standardised protocol and procedures.

The data collected via questionnaire, will include the following: socio-demographic information, personal and family medical history, dietary data using a quantified food frequency questionnaire and a single quantified 24-hour recall, physical activity (Global physical activity questionnaire – GPAQ) [21] and physical environment (neighbourhood environment walkability scale (NEWS) Africa) [22]. Anthropometric and BP measurements will be repeated using the standardised techniques described above. Questionnaire information will be captured electronically on REDCap (a secure web application for building and managing online surveys and databases) using a password protected personal digital assistant (PDA). Built-in checks will allow real-time quality control of the data at the point of collection. The same measurements will be completed at follow-up at 12 months. Refer Table 2.

**Table 2:** Measurements and tools for data collection for the Eastern Cape African Diabetes Prevention Programme

Variable	Component	Measurements tools/questions
**Socio-demographic measures**		Age, gender, area, community, current marital status, education level, occupation, income
**Behavioural measures**	Tobacco use	WHO STEPS questionnaire [15]
	Alcohol use	WHO STEPS questionnaire [15]
	Sedentary behaviour	Time spent in front of a screen
	Sleep	Time, quality
**Psychological measures**	Chronic stress	Chronic stress scale [14]
**Physical activity measures**	Physical activity pattern	WHO STEPS questionnaire: global physical activity questionnaire (GPAQ) [15]
	Barriers to physical activity	Scale adapted from the one designed by Booth et al. [16]
	Self-efficacy	Scale adapted from the exercise self-efficacy scale (ESES) designed by Schwarzer and Jerusalem [17]
**Medical history**		Family history of diabetes
**Clinical measures**	Waist circumference	Measured between the lower border of the lowest rib and upper border of the iliac crest/pelvic bone to the nearest 0.1 cm.
	Weight	Weight measurement with minimal clothing on a digital (SECA) scale, recorded to the nearest 0.1 kg
	Height	Standing height, minimal clothing, aligning head in a standard anatomical position using a SECA stadiometer
	SBP	Electronic M6 COMFORT OMRON device with an integrated cuff
	DBP HbA1c	Electronic M6 COMFORT OMRON device with an integrated cuff HbA1c measured using fasting blood and HPLC
**Neighbourhood indicators**	Stores and facilities, Access to services and places, Roads and walking paths, places for walking/cycling/playing, Surroundings, Safety from crime and traffic, Personal safety, Stranger danger	Neighbourhood Environment Walkability Scale (NEWS) Africa Questionnaire [18]

SBP = systolic blood pressure; DBP = diastolic blood pressure; HbA1c = glycosylated haemoglobin; HPLC = High-performance liquid chromatography; WHO = World Health Organisation. [Adapted from Hill et al. 2020 [12]].

#### Intervention monitoring and evaluation

Process evaluation of the programme is both formative and summative, to serve the dual purpose of aiding the modification of the intervention during the implementation phase, and to assist in evaluating the suitability, applicability, and appropriateness of the intervention. Targeted elements of the implementation will include the fidelity, the dose (delivered and received), the reach, recruitment, retention/maintenance, and context. The PIPE-4S framework [23] will be used to evaluate the intervention. Indicators and measures relevant to each dimension of the framework will be collected. Information will also be collected from group facilitators and participants regarding the feasibility, acceptability, and satisfaction with the programme and its delivery. For this purpose, we will use intervention logs, facilitator notes and participant evaluation sheets during the period of intervention. Post-intervention, copies will be made of the participant workbooks which would include the goals they set, goal tracking and any notes that they may have included. Focus group discussions will be held post follow-up to engage with participants about their experiences through the intervention and into the maintenance phase. Facilitators and barriers during the process will be discussed, as well as any suggestions on improvement of the programme. Furthermore, we would like to investigate both the (possible) personal and familial benefits the intervention programme enabled. We aim to conduct a minimum of eight focus group sessions at the intervention sites, with the schedules developed accordingly.

The reach of the intervention to its intended audience will be assessed by comparing the characteristics of eligible participants who did or did not proceed to participate in 1) screening, b) baseline measurement, and c) the group programme.

#### Economic analysis and implementation measurement

The direct medical costs and cost-effectiveness of the intervention will be assessed. The relative cost will be estimated from the health system perspective, and costs of diabetes case detection and case prevention will be evaluated. Incremental cost-effectiveness will be calculated based on the risk reduction achieved [24]. Productivity and financial gains for individuals/country from fewer workdays missed will be assessed.

### Data analyses

#### Quantitative data analyses

Analyses will be conducted at baseline and 12-month follow-up. Analysis will be conducted overall and by subgroups defined by gender and age (above vs. below median age). These subgroups analysis will however serve mostly to confirm the homogeneity of the main results since the study has not been powered to detect significant results at the subgroup level. The characteristics of participants selected, screened, and included in the study will be summarised by cluster. We will then report the number of participants excluded at each step and the number of participants meeting the inclusion criteria. Baseline analyses will then investigate differences between clusters and other major subgroups that are likely to influence in a differential way the outcome of the intervention. This will be done with the use of chi square test and equivalent for qualitative characteristics, and Student t-test, ANOVA, and equivalents.

Analysis during follow-up will determine the extent that the programme goals were achieved through overall and subgroup analysis. Adherence to the intervention will be assessed by estimating the number of face-to- face intervention sessions effectively attended by individuals.

An analysis of covariance approach that adjust for individual and cluster differences (Klar and Darlington [25] at baseline will be conducted. This could be achieved using Generalised estimating equations (GEE) to model the effect of the intervention. A small-sample correction analyses will be considered to maintain the type I error rate at or below 5%. Important independent variables to be considered will be the cluster and the indicator of intervention exposure for each. Individual level characteristics to be included in the models will be pre-specified. Population average models as opposed to random effect models (also known as marginal models) will be used. Within the framework of cluster randomised trials (CRTs), random effects models lack both appropriate interpretation and might be biased. Analysis of the secondary outcome will take a form similar to that for the primary outcome. For both primary and secondary outcome, in addition to GEE, we will consider the Generalised Linear Mixed Model (GLMM) approach [26]. Quantitative data analysis will use the R statistical software (The R Foundation) and relevant packages (i.e. SAS).

Since missing values are unavoidable, these values will be handled with extra caution using appropriate statistical techniques. Reasons and mechanisms for the missing data points will be explored by various summary statistics as well as graphical displays.

#### Qualitative data analyses

All qualitative data collected will be analysed by thematic analysis using a reciprocal coding approach, where researchers engage in open dialogue about themes and data interpretation. The analysis will be aided by Atlas ti.7, a qualitative data management and analysis tool. The theoretical framework underpinning the qualitative analysis is constructivist insofar as interpretation will consider the multiplicities of views informing participants’ experiences. Transcripts (field notes, debriefing sessions, and interviews) will be first reviewed independently, and then through dialogue, and composite themes will be developed by at least two researchers. Thematic analysis, or pattern coding, is a method for grouping diverse sections of data into smaller analytic units. A coding framework will be developed.

## Time line

The timeline for this project is 24 months, refer Table 3 below:

**Table 3.**
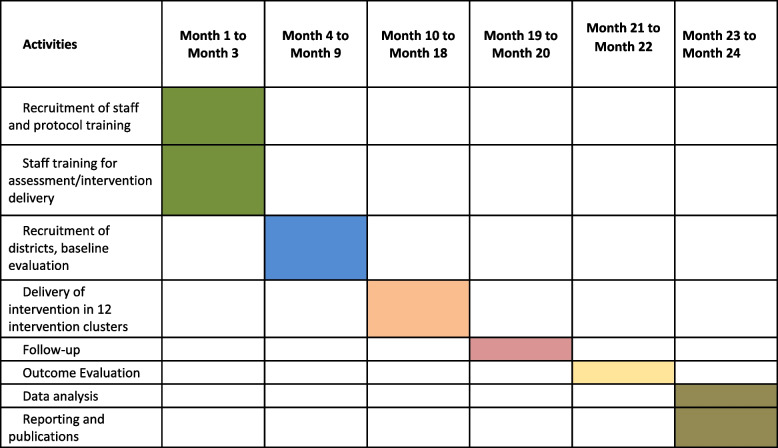
Project timeline

## Discussion

The principal purpose of the current study is to evaluate the implementation and suitability of the SA-DPP to peri-urban/rural and previously disadvantaged (arguably still disadvantaged) communities in the Eastern Cape Province of South Africa. This program was developed and tailored in urban populations in the Western Cape Province. To date the development and adaption of the SA-DPP has been focused on black and mixed-ancestry individuals residing in poorly resourced communities in Cape Town and applicability to the rest of SA needs to be established. We propose to implement and evaluate a DPP that has been adapted for SA [12, 27, 28], which is based on interventions previously shown to be effective in Finland [6, 29] and Australia [7], and is currently been trialed in other developing countries, such as India [30].

The knowledge accumulated in implementing DPPs around the world over the past two decades, clearly shows that implementing a DPP in a new setting almost always translate into a program adaption or new program [25, 31]. This “new program” would be context and culturally appropriate. Therefore, our proposed Eastern Cape Diabetes Prevention Program (EC-DPP) cannot be conceptualized as a simple expansion of the program developed and tailored in urban populations in the Western Cape Province. Critical analyses of the qualitative feedback from EC-DPP participants, and the impact of the program on their lifestyle (i.e. quality of life, dietary and physical activity behavioural changes) and financial status will generate a unique knowledge that will guide the adoption and application of a uniform policy for primary prevention of T2DM at primary health care level in diverse populations of SA. Alternatively, the data set may be unique to an extent of adjusting the program to suit only a certain group of SAs.

The processes and results of this study will lead to a model for the prevention of diabetes and other diseases of lifestyles in SA and other countries in the region.

## Supplementary Information

**Additional file 1.** Intervention Components of Finnish, Australian and Indian DPP, tabulated literature

**Additional file 2.** OR Tambo District Munciaplity Map, Map

**Additional file 3.** Information sheet and consent forms, Information sheet and consent forms

**Additional file 4.** Project Team, profile and roles of the project team

## Data Availability

When results at the end of the intervention show a significant effect of the intervention on outcomes such as body weight, we will start preparing a report for the Department of Health (DoH) to own the programme and implement it in their pilot health districts, and subsequently in other districts. We will help prepare the training package and instruments for the delivery of SA-DPP at primary healthcare level, which will then be incorporated by the DoH as a module in the training curriculum for non-professional health workers developed by the DoH.

## Abbreviations

SA-DPP: The South African Diabetes Prevention Programme
SA: South Africa
NPHW: non-professional health workers
OGTT: oral glucose tolerance test
BMI: body mass index
T2DM: type 2 diabetes mellitus
NCDRU: Non-Communicable Diseases Research Unit
ADRS: African Diabetes Risk Score
DPS: Diabetes Prevention Study
ICC: intra-cluster correlation coefficient
GPAQ: Global physical activity questionnaire
NEWS: neighbourhood environment walkability scale
PDA: personal digital assistant
GEE: Generalized estimating equations
CRT: cluster randomized trial
GLMM: Generalised Linear Mixed Model
DPP: diabetes prevention program
EC-DPP: Eastern Cape Diabetes Prevention Program
DoH: Department of Health
