# Supplementary material for: A study protocol for a cluster randomized controlled trial to test the applicability of the South African diabetes prevention program in the Eastern Cape Province of South Africa
Source: BMC Public Health. 2023 Jan 31;23:214. doi: 10.1186/s12889-022-14884-1 (PMC9890849; doi:10.1186/s12889-022-14884-1)
Supplement: Supplementary file 1 — Additional file 1. Intervention Components of Finnish, Australian and Indian DPP, tabulated literature [file 12889_2022_14884_MOESM1_ESM.docx]

Additional file1:

Table 1: Intervention Components of Finnish, Australian and Indian DPP

| **Article/s** | **Programme objectives - outcomes** | **Duration** | **Intervention format** | **Intervention/curriculum topics** |
| --- | --- | --- | --- | --- |
| **FINNISH DPP**   1. Absetz P, Valve R, Oldenburg B, Heinonen H, Nissinen A, Fogelholm M, et al. Type 2 diabetes prevention in the "real world": one-year results of the GOAL Implementation Trial. Diabetes Care. 2007;30(10):2465-70 2. Tuomilehto, J., Lindström, J., Eriksson, J.G., Valle, T.T., Hämäläinen, H., Ilanne-Parikka, P., Keinänen-Kiukaanniemi, S., Laakso, M., Louheranta, A., Rastas, M. and Salminen, V., 2001. Prevention of type 2 diabetes mellitus by changes in lifestyle among subjects with impaired glucose tolerance. *New England Journal of Medicine*, *344*(18), pp.1343-1350. 3. Uutela, A., Absetz, P., Nissinen, A., Valve, R., Talja, M. and Fogelholm, M., 2004. Health Psychological Theory in Promoting Population Health in Paijat-Hame, Finland: First Steps toward a Type 2 Diabetes Prevention Study. *Journal of Health Psychology*, *9*(1), pp.73-84. | Content and design of the intervention was underpinned by the five key life-style change objectives that were the focus of the DPS:    1. Less than 30% of total energy intake from fat;  2. Less than 10% of total energy intake from saturated fat;  3. At least 15 g of fiber/1,000 kcal;  4. At least 4 h/week moderate level physical activity; and  5. More than 5% weight reduction.  Attainment of at least four of these objectives was sufficient to prevent type 2 diabetes | 8 months:  first 5 sessions extended over 8 weeks, with 2-week intervals; last session at 8 months | 1. The programme consists of six two-hour sessions facilitated by trained public health nurses, diabetes nurses and/or physiotherapists. 2. A dietician participates in each group during one session, and in another session the group visits municipal sports and recreation facilities where possibilities for leisure time physical activity are presented. 3. The programme uses group approach based on empowerment ideology, emphasizing the participants’ possibilities to make informed choices, and his/her role as an independent decision-maker who takes responsibility and regulates his/her own actions. | **Session 1**   - Learning to know each other - Rules for the group - Discussion on current beliefs: how does life-style influence health? - Introduction by the facilitator: diabetes, risk factors & development, effects, prevention - Reflective discussion and re-evaluation of beliefs - Exercise: Dream—where do we want to be in 12 months’ time? - How to make the dream come true: goals, planning, homework and other exercises - Homework assignments: monitoring own behaviour with food diary and physical activity schedule   **Session 2**   - Returning of food diaries - Introduction by the facilitator: prevention really works - Evaluating own behaviour: feedback from physical activity schedule, fibre and fat tests - Discussion in small groups: comparison of own habits with the diet and physical activity goals sufficient for prevention - Role model stories with features contributing to success/failure - Discussion: analysis and re-attribution of previous successful/unsuccessful experiences - Homework assignments: preparation for goal setting, monitoring physical activity and eating habits - Discussion: barriers for group work and participation   **Session 3**   - Feedback from the physical activity schedule - Introduction by the facilitator: health effects of physical activity - Goal planning: - Discussion: are the selected goals concrete, positive, attainable, developing? - Individual task: short-term (immediate) Where, When, How, ‘equipment’ - Feedback from homework: difficult & easy situations, what to do? - Goal setting - Homework assignments: feedback and re-inforcement; monitoring physical activity and eating habits - Possibilities for physical activity in the local community: presentation of choices and facilities   **Session 4**   - Food choices: feedback based on findings from food diaries - Introduction by the dietician: how to eat healthy? - Goal planning:   — Discussion: are the selected goals concrete, positive, attainable, developing?  — Individual task: short term (immediate) Where, When, How, ‘equipment’   - Feedback from homework: difficult & easy situations, what to do? - Goal setting - Exercise: how to make one’s favorite food/dishes lighter? - Homework assignments: positive feedback in getting social support; monitoring physical activity and eating habits   **Session 5**   - Discussion: evaluating and refining the goals - Discussion: routines—have they already changed? Physical activity schedule, fibre and fat tests - Individual task: intermediate goals (next 6 months) - Exercise: how to overcome barriers, how to use resources in maintaining the behaviour changes - Discussion of ways to create peer group support system - Homework assignments: monitoring physical activity and eating habits   **Session 6**   - Discussion: evaluating the goals - Discussion: routines—have they already changed? Physical activity schedule, fibre and fat tests - Group discussion: analysis and re-attribution of success and failure - Discussion: future goals - Discussion: evaluation of the group work |
| **AUSTRALIAN DPP**   1. Laatikainen, T., Dunbar, J.A., Chapman, A., Kilkkinen, A., Vartiainen, E., Heistaro, S., Philpot, B., Absetz, P., Bunker, S., O'Neil, A. and Reddy, P., 2007. Prevention of type 2 diabetes by lifestyle intervention in an Australian primary health care setting: Greater Green Triangle (GGT) Diabetes Prevention Project. *BMC public health*, *7*(1), pp.1-7. 2. Kilkkinen, A., Heistaro, S., Laatikainen, T., Janus, E., Chapman, A., Absetz, P. and Dunbar, J., 2007. Prevention of type 2 diabetes in a primary health care setting: Interim results from the Greater Green Triangle (GGT) Diabetes Prevention Project. *Diabetes research and clinical practice*, *76*(3), pp.460-462. 3. Uutela, A., Absetz, P., Nissinen, A., Valve, R., Talja, M. and Fogelholm, M., 2004. Health Psychological Theory in Promoting Population Health in Paijat-Hame, Finland: First Steps toward a Type 2 Diabetes Prevention Study. *Journal of Health Psychology*, *9*(1), pp.73-84. | 1. no more than 30% of energy from fat;  2. no more than 10% of energy from saturated fats;  3. at least 15 g/1000 kcal fibre;  4. at least 30 min/day moderate physical activity;  5. at least 5% weight reduction | 8 months:  6 structured 90-minute group sessions over 8 months   - The first five sessions occurred within the first three months, with two week intervals between sessions. The last session took place at eight months. | 1. The sessions were facilitated by specially trained study nurses, dietitians and physiotherapists. A goal setting approach was used to motivate individuals to progress from intention to actual behaviour change. Regular self-assessment was used to empower participants to take responsibility for their own decisions and to make informed choices. 2. Social support was enhanced by the group setting and by encouraging participants to seek support from their own social networks. | ‘The intervention model used in the study was based on the diabetes prevention project in the Finnish GOAL study’  **Session 1**   - Learning to know each other - Rules for the group - Discussion on current beliefs: how does life-style influence health? - Introduction by the facilitator: diabetes, risk factors & development, effects, prevention - Reflective discussion and re-evaluation of beliefs - Exercise: Dream—where do we want to be in 12 months’ time? - How to make the dream come true: goals, planning, homework and other exercises - Homework assignments: monitoring own behaviour with food diary and physical activity schedule   **Session 2**   - Returning of food diaries - Introduction by the facilitator: prevention really works - Evaluating own behaviour: feedback from physical activity schedule, fibre and fat tests - Discussion in small groups: comparison of own habits with the diet and physical activity goals sufficient for prevention - Role model stories with features contributing to success/failure - Discussion: analysis and re-attribution of previous successful/unsuccessful experiences - Homework assignments: preparation for goal setting, monitoring physical activity and eating habits - Discussion: barriers for group work and participation   **Session 3**   - Feedback from the physical activity schedule - Introduction by the facilitator: health effects of physical activity - Goal planning: - Discussion: are the selected goals concrete, positive, attainable, developing? - Individual task: short-term (immediate) Where, When, How, ‘equipment’ - Feedback from homework: difficult & easy situations, what to do? - Goal setting - Homework assignments: feedback and re-inforcement; monitoring physical activity and eating habits - Possibilities for physical activity in the local community: presentation of choices and facilities   **Session 4**   - Food choices: feedback based on findings from food diaries - Introduction by the dietician: how to eat healthy? - Goal planning:   — Discussion: are the selected goals concrete, positive, attainable, developing?  — Individual task: short term (immediate) Where, When, How, ‘equipment’   - Feedback from homework: difficult & easy situations, what to do? - Goal setting - Exercise: how to make one’s favorite food/dishes lighter? - Homework assignments: positive feedback in getting social support; monitoring physical activity and eating habits   **Session 5**   - Discussion: evaluating and refining the goals - Discussion: routines—have they already changed? Physical activity schedule, fibre and fat tests - Individual task: intermediate goals (next 6 months) - Exercise: how to overcome barriers, how to use resources in maintaining the behaviour changes - Discussion of ways to create peer group support system - Homework assignments: monitoring physical activity and eating habits   **Session 6**   - Discussion: evaluating the goals - Discussion: routines—have they already changed? Physical activity schedule, fibre and fat tests - Group discussion: analysis and re-attribution of success and failure - Discussion: future goals   Discussion: evaluation of the group work |
| **INDIAN DPP**   1. Thankappan KR, Sathish T, Tapp RJ, Shaw JE, Lotfaliany M, Wolfe R, et al. A peer-support lifestyle intervention for preventing type 2 diabetes in India: A cluster-randomized controlled trial of the Kerala Diabetes Prevention Program. PLoS medicine. 2018;15(6):e1002575. 2. Daivadanam, M., Absetz, P., Sathish, T., Thankappan, K.R., Fisher, E.B., Philip, N.E., Mathews, E. and Oldenburg, B., 2013. Lifestyle change in Kerala, India: needs assessment and planning for a community-based diabetes prevention trial. *BMC Public Health*, *13*(1), pp.1-16. 3. Absetz P, Valve R, Oldenburg B, Heinonen H, Nissinen A, Fogelholm M, et al. Type 2 diabetes prevention in the "real world": one-year results of the GOAL Implementation Trial. Diabetes Care. 2007;30(10):2465-70 | 1. Increasing physical activity 2. Promoting healthy eating habits 3. Maintaining appropriate body weight by balancing calorie intake and physical activity 4. Tobacco cessation 5. Reducing alcohol consumption 6. Ensuring adequate sleep | 12 months:  15 group sessions | an introductory session delivered by the DPP team; two education sessions conducted by local experts; and 12 sessions delivered by trained lay peer leaders. | ‘The intervention model used in the study was based on the diabetes prevention project in the Finnish GOAL study’  **Session 1**   - Learning to know each other - Rules for the group - Discussion on current beliefs: how does life-style influence health? - Introduction by the facilitator: diabetes, risk factors & development, effects, prevention - Reflective discussion and re-evaluation of beliefs - Exercise: Dream—where do we want to be in 12 months’ time? - How to make the dream come true: goals, planning, homework and other exercises - Homework assignments: monitoring own behaviour with food diary and physical activity schedule   **Session 2**   - Returning of food diaries - Introduction by the facilitator: prevention really works - Evaluating own behaviour: feedback from physical activity schedule, fibre and fat tests - Discussion in small groups: comparison of own habits with the diet and physical activity goals sufficient for prevention - Role model stories with features contributing to success/failure - Discussion: analysis and re-attribution of previous successful/unsuccessful experiences - Homework assignments: preparation for goal setting, monitoring physical activity and eating habits - Discussion: barriers for group work and participation   **Session 3**   - Feedback from the physical activity schedule - Introduction by the facilitator: health effects of physical activity - Goal planning: - Discussion: are the selected goals concrete, positive, attainable, developing? - Individual task: short-term (immediate) Where, When, How, ‘equipment’ - Feedback from homework: difficult & easy situations, what to do? - Goal setting - Homework assignments: feedback and re-inforcement; monitoring physical activity and eating habits - Possibilities for physical activity in the local community: presentation of choices and facilities   **Session 4**   - Food choices: feedback based on findings from food diaries - Introduction by the dietician: how to eat healthy? - Goal planning:   — Discussion: are the selected goals concrete, positive, attainable, developing?  — Individual task: short term (immediate) Where, When, How, ‘equipment’   - Feedback from homework: difficult & easy situations, what to do? - Goal setting - Exercise: how to make one’s favorite food/dishes lighter? - Homework assignments: positive feedback in getting social support; monitoring physical activity and eating habits   **Session 5**   - Discussion: evaluating and refining the goals - Discussion: routines—have they already changed? Physical activity schedule, fibre and fat tests - Individual task: intermediate goals (next 6 months) - Exercise: how to overcome barriers, how to use resources in maintaining the behaviour changes - Discussion of ways to create peer group support system - Homework assignments: monitoring physical activity and eating habits   **Session 6**   - Discussion: evaluating the goals - Discussion: routines—have they already changed? Physical activity schedule, fibre and fat tests - Group discussion: analysis and re-attribution of success and failure - Discussion: future goals   Discussion: evaluation of the group work |
