# Supplementary figures and images for: A study protocol for a cluster randomized controlled trial to test the applicability of the South African diabetes prevention program in the Eastern Cape Province of South Africa
Source: BMC Public Health. 2023 Jan 31;23:214. doi: 10.1186/s12889-022-14884-1 (PMC9890849; doi:10.1186/s12889-022-14884-1)

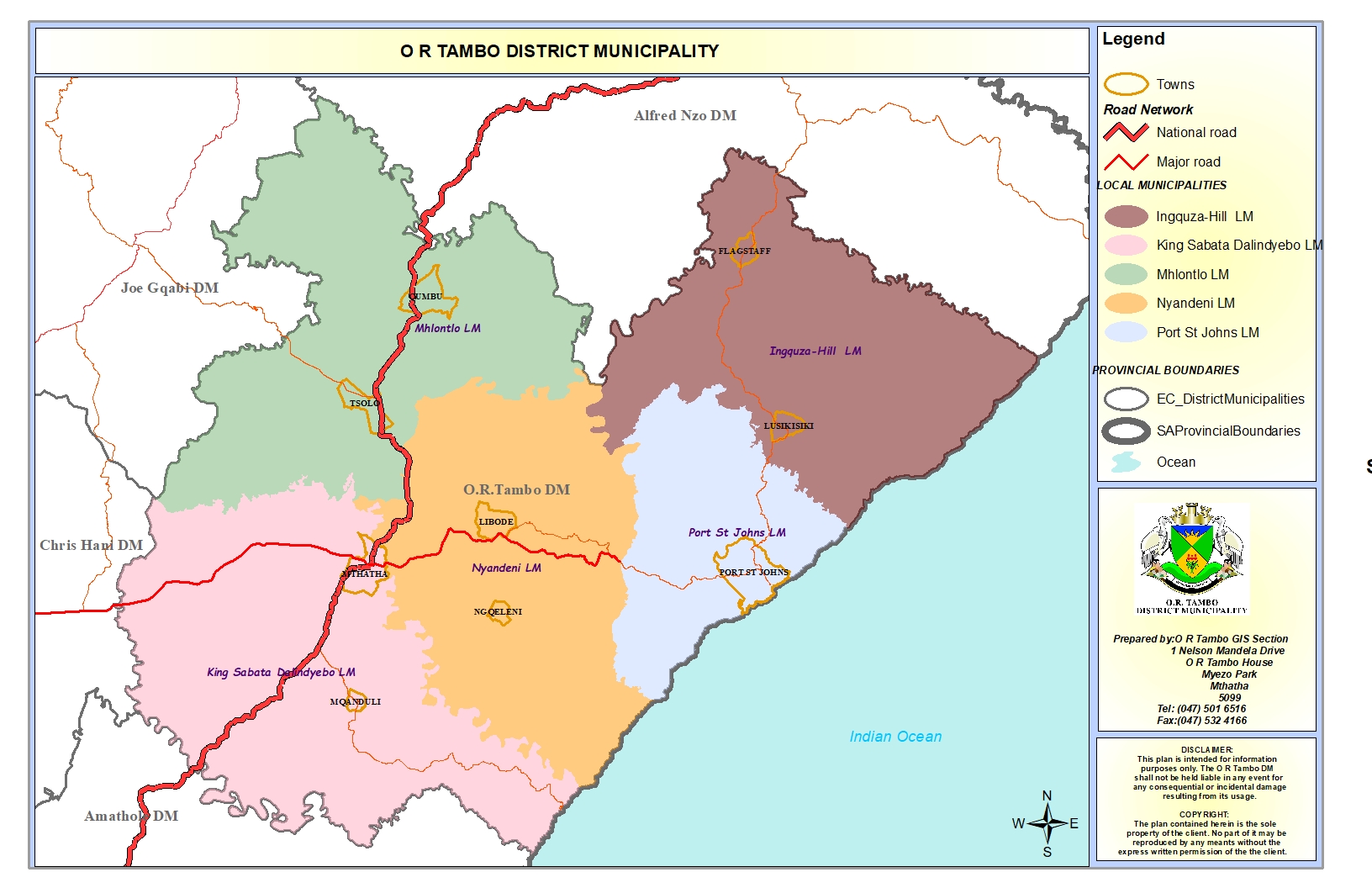

Supplement: Supplementary file 2 — Additional file 2. OR Tambo District Munciaplity Map, Map [file 12889_2022_14884_MOESM2_ESM.jpg]
