# Supplementary material for: A study protocol for a cluster randomized controlled trial to test the applicability of the South African diabetes prevention program in the Eastern Cape Province of South Africa
Source: BMC Public Health. 2023 Jan 31;23:214. doi: 10.1186/s12889-022-14884-1 (PMC9890849; doi:10.1186/s12889-022-14884-1)
Supplement: Supplementary file 3 — Additional file 3. Information sheet and consent forms, Information sheet and consent forms [file 12889_2022_14884_MOESM3_ESM.pdf]

CONSENT TO PARTICIPATE IN A RESEARCH STUDY
STUDY TITLE: The Eastern Cape Diabetes Prevention Programme (EC-DPP)
Principal investigators:

Jillian Hill, (PhD)
Senior Scientist: Non-communicable Diseases Research Unit (NCDRU), South African Medical Research Council
Yandiswa Yako, (PhD)
Senior Lecturer, Department Human Biology, Faulty of Health Sciences, Walter Sisulu University

Dear Sir/Madam,
We are scientists from the South African Medical Research Council (MRC) and Walter Sisulu University. We would like to conduct a study to prevent the development of diabetes in your community.

Purpose and focus of the study
Diabetes is becoming more common than before and we want to try to decrease the risk of people in the community developing the disease. We hope to do this by inviting people at high risk for diabetes, detected after blood testing, to participate in a diabetes prevention programme. The people invited to participate in the study will be educated about the condition and on how to decrease their risk for diabetes. This will be done in 6 group discussions, lasting about 2 hours each. The first 5 sessions will be held every 2 weeks over 2 months and the last session will be 6 months later. Trained community healthcare workers will conduct the group discussions at a time most convenient for members of the group. They will also send text messages over from the beginning of the intervention for nine months to people in these groups for support.

Before we can do this, we will need to identify people at high risk for diabetes. We will do this in the following way:

1. Questionnaires: We will first identify people at high risk for diabetes by asking them a few questions about their general and medical history.
2. Blood sampling: People identified as high risk will undergo testing to see whether they have diabetes or are at high risk for diabetes. People with diabetes will be referred and given a referral letter for their nearest clinic or doctor.

Who can take part in the study?
The following steps will be used to identify people who can take part in the intervention study:
1) People between the ages of 25-65 years and not known to have diabetes will be screened to check their risk for diabetes.
2) After screening, people thought to be at high risk for diabetes will be invited to undergo an oral glucose tolerance test where blood samples will be drawn to examine for diabetes.
3) Thereafter, people found to be at high risk for diabetes on blood testing but who did not have diabetes, will be invited to participate in the intervention study to reduce their risk for diabetes.
4) Half the people invited to participate in this study will attend the group sessions while the other half will receive written information about healthy lifestyle and lowering diabetes risk. The allocation into these 2 groups will be done randomly by a statistician on the computer. At the end of the study, people who were not in the discussion group will be invited to also attend these sessions.

Who will have their blood taken?
People who are identified as being at high risk for diabetes on the screening questions will be invited to undergo blood testing.

People who are identified at high risk for diabetes on blood testing and are included in the study will have their blood tested again 1 year later.

What tests will be done?
The blood tests will include an oral glucose tolerance test to diagnose diabetes and is described below. The other tests include tests to check for high cholesterol (fat) in the blood, for heart and kidney problems and for tobacco use. These tests show if a person is at risk for a heart attack or stroke. We will store any blood that remains for testing later. This blood will be stored in an ultralow temperature freezer at the MRC for up to 20 years. The Research Ethics Committee of the MRC will approve any future tests.

Oral glucose tolerance test
After an overnight 10 hour fast (where you do not eat or drink anything except water after your evening meal, the night before the blood sampling, and miss your breakfast the day of the test), we will place a sterile little tube in a vein in your arm and take 15 mls (3 teaspoons) of blood from this. We will give you a cup of sugar water to drink and then we will take two more blood samples (each 2 teaspoons) over the next two hours. Taking the blood sample may cause a little discomfort at the site but there are no risks for this test, other than those associated with routine blood sampling. All procedures will be supervised and carried out by appropriately trained medical personnel who will use techniques to minimise any risks of infection. This test is used routinely for medical purposes. The blood sample will be used to determine your blood sugar, insulin, cholesterol and other additional factors that may help us learn more about diabetes.

Do I have to take part in the study?
You have no obligation to participate in the study and you may withdraw from the study at any time. There will be no penalty to you if you decide not to participate in the study, or if you want to withdraw from the study later on. Remember: Your participation in this study is completely voluntary.
If you decide to withdraw from the study, you may be asked why you have decided to withdraw for statistical purposes but giving reasons for withdrawal from the study is also completely voluntary.

What can I expect to gain from participating in this study?
1. You will have access to trained staff who will check your risk for diabetes.
2. If your blood is tested or blood pressure measurements taken, you will receive copies of your blood tests and measurements for your records.
3. You will be referred for appropriate treatment if any abnormalities are found
4. You will contribute to medical research that may provide very useful insights on how to prevent diabetes

You will receive at no cost to yourself, an assessment of your risk for diabetes. For people at high risk, you will receive free testing to check if you have diabetes.

What will be the costs of my participation in this study?
1. You will need to provide your own transport to and from the research facility/clinic for blood testing if you are found to have high risk, but you will be reimbursed a Shoprite money voucher for your time and transport costs. This will be for R100-00 for each visit when blood tests are done, and questionnaires administered because you will need to travel to the study venue. The voucher will be for R50 for each visit when you attend the group sessions, will be held in the community, where you learn more on how to prevent diabetes.
2. You will be required to sacrifice your time for completion of questionnaires, group discussion session and medical assessments/testing. The questionnaire and blood tests, to be done 2 times in total, at yearly intervals, will be about 1-2 hours long. The 6 groups sessions will each be 2 hours long.

What will happen to the data collected during the study?
The data collected will be sent to researchers at the South African Medical Research Council and Walter Sisulu University and used solely for this study.

Who will know that I participated in this study?

Only the research staff and other participants in the study will know that you participated in the study. Your name will not be recorded or revealed to anyone not involved in the study, and your name or other details will not be published in any documents.

Will I be informed about the results of the study?
You will be informed about the study results after publication.

How will I find out about my results?
A member of the research team will contact you directly by telephone and advise you or refer you for further medical assistance if you are diagnosed with diabetes. Your blood results will be sent to you by post as soon as they become available. People with high risk will be informed of their results and invited to continue with the study.

What are my rights while taking part in the study?
Your taking part in this study is your choice and you are free to not take part. All information will be confidential and anonymous. You may also refuse to answer any questions you do not feel comfortable answering and you may stop during the interview and not continue. Your name will not be linked to the information collected at any time and will not appear in any report or publication.

What are the risks to my health in this study?
Your participation in this study will involve answering questions, and for those at high risk, it will include a medical examination and collection of blood for laboratory analysis.
Blood will be collected by an experienced professional nurse, doctor or appropriately trained staff member. Having your blood drawn can cause bleeding, bruising and in rare cases an infection may occur at the site of the needle stick and may also be uncomfortable. Rarely, light-headedness or fainting may occur.

If you fall ill, suffer any side effects or if you are injured in any study related manner contact the investigator/researcher immediately. The MRC, as the sponsor of this study, has taken out the necessary insurance to cover you as a research participant.

This study has been approved by the Research Ethics Committee of the Medical Research Council, Cape Town and will be carried out according to the ethical guidelines and principles of the International Declaration of Helsinki, 2013. If you have questions about your rights as someone who took part in the study, you are welcome to contact the Chairperson of the Research Ethics Committee, Prof Danie Du Toit, at the Medical Research Council, P.O. Box 19070, Tygerberg. 7505. Contact telephone number: 021 938 0687; email: adri.labuschagne@mrc.ac.za. You will receive a copy of this information sheet and consent form for your own records. . If you have any questions or concerns about the research, please feel free to contact Dr Jillian Hill (021 938 0811) at the Medical Research Council, Tygerberg, Cape Town.

PLACE BARCODE STICKER HERE

Declaration by the participant
By signing this form, I ………………………………………………………………………………………..

Initial those that you agree to

1. Agree to take part in a research study titled: The Eastern Cape Diabetes Prevention Programme (EC-DPP) ....
2. Agree to have my blood taken and tested ....
3. Agree to have my blood samples stored (If you decide at any time to withdraw this consent you can contact the MRC and asked for your blood sample to be destroyed) ....
4. Agree that the research steam may contact me in the future for a follow-up study ....

I declare that:

- I have read or had read to me this information and consent form and it is written in a language with which I am fluent and comfortable.
- I am older than 25 years of age
- I have had a chance to ask questions and all my questions have been adequately answered.
- I know that taking part in this study is voluntary and l have not been forced to take part. I may choose to leave the study at any time without any problems.

Signed at:	______________	on (date)	______________
Name of participant	______________	Signature of participant	______________
Name of witness	______________	Signature of witness	______________

Declaration by investigator (or person designated)
I (name) .... declare that I have explained the information in this document to …………………………………………… I have encouraged him/her to ask questions and took adequate time to answer them. I am satisfied that he/she adequately understands all aspects of the research, as discussed above.

Signed at:	______________	on (date)	______________

Name of investigator		Signature of investigator	

CONSENT TO PARTICIPATE IN A RESEARCH STUDY
STUDY TITLE: The Eastern Cape Diabetes Prevention Programme (EC-DPP)
Principal investigators:

Jillian Hill, (PhD)
Senior Scientist: Non-communicable Diseases Research Unit (NCDRU), South African Medical Research Council
Yandiswa Yako, (PhD)
Senior Lecturer, Department Human Biology, Faulty of Health Sciences, Walter Sisulu University

Dear Sir/Madam,

We are scientists from the South African Medical Research Council. Diabetes is becoming more common than before and we want to try to decrease the risk of people in the community developing the disease. We would like to take a blood sample to examine the genes in your blood related to diabetes and cardio-metabolic diseases. This is a new area of study and not all genetic aspects of diabetes and cardio-metabolic diseases are currently known.

Genes contains all the information about our physical characteristics, such as how we look, the colour of our eyes or how tall we will grow. These characteristics are passed down through the genes to us from our parents and it is present in all the cells in our body. For example, the genes in our blood has information on diseases we may inherit from our parents such as diabetes or heart disease. We would like to test for genes linked to diabetes and see whether the risk for diabetes can be decreased even when the diabetes genes are present.

These tests are done anonymously and will not be linked back to any specific person. DNA will be isolated and stored for future analyses of genes related to diabetes and cardio-metabolic diseases. This blood will be stored in an ultralow freezer for up to 20 years. The Research Ethics Committee of the Medical Research Council will approve any future tests.

You have no obligation to agree for your blood to be taken and stored for genetic studies. You may ask for your blood samples back or to be destroyed at any time. There will be no penalty to you if you decide to withdraw your permission later. Remember: Your participation in this study is completely voluntary.

This study has been approved by the Research Ethics Committee of the Medical Research Council, Cape Town and will be carried out according to the ethical guidelines and principles of the International Declaration of Helsinki, 2013. If you have questions about your rights as someone who took part in the study, you are welcome to contact the Chairperson of the Research Ethics Committee, Prof Danie Du Toit, at the Medical Research Council, P.O. Box 19070, Tygerberg. 7505. Contact telephone number: 021 938 0687; email: adri.labuschagne@mrc.ac.za. You will receive a copy of this information sheet and consent form for your own records. . If you have any questions or concerns about the research, please feel free to contact Dr Jillian Hill (021 938 0345) at the Medical Research Council, Tygerberg, Cape Town.

I declare that:

- I have read or had read to me this information and consent form and it is written in a language with which I am fluent and comfortable.
- I am older than 18 years of age
- I have had a chance to ask questions and all my questions have been adequately answered.
- I know that taking part in this study is voluntary and I have not been forced to take part. I may choose to leave the study at any time without any problems.

Signed at:	______________________	on (date)	______________________
Name of participant	______________________	Signature of participant	______________________
Name of witness	______________________	Signature of witness	______________________

Declaration by investigator (or person designated)

I (name) .... declare that I have explained the information in this document to ……………………………………………. I have encouraged him/her to ask questions and took adequate time to answer them. I am satisfied that he/she adequately understands all aspects of the research, as discussed above.

Signed at:	______________________	on (date)	______________________
Name of investigator	______________________	Signature of investigator	______________________
