# Supplementary material for: A study protocol for a cluster randomized controlled trial to test the applicability of the South African diabetes prevention program in the Eastern Cape Province of South Africa
Source: BMC Public Health. 2023 Jan 31;23:214. doi: 10.1186/s12889-022-14884-1 (PMC9890849; doi:10.1186/s12889-022-14884-1)
Supplement: Supplementary file 4 — Additional file 4. Project Team, profile and roles of the project team [file 12889_2022_14884_MOESM4_ESM.docx]

## **Project Team**

## **1.1 Roles and responsibilities:**

| **Name and Surname** | | **Institution** | **Role in Project** |
| --- | --- | --- | --- |
| Dr Jillian Hill | | South African Medical Research Council | Principal investigator: Dr Hill has overseen all aspects of the successful development of SA-DPP and is best positioned to guide the successful adaptation and implementation in Eastern Cape. She will be involved in all aspects of training, SOP development and intervention refinement. She will provide support to Dr Yako who will be the onsite project manager. |
| Dr Yandiswa Yako | | Walter Sisulu University | Principal investigator: Dr. Yako is an academic researcher at Walter Sisulu University, which is situated in the OR Tambo district where recruitment will be conducted. She is currently involved in a project that screens participants residing in the OR Tambo, Chris Hani, and Alfred Ndzo district for NCDs such as T2D, hypertension, and dyslipidemia. She acquired other necessary skills of conducting community-based research project from the Bellville South Study, which was used for adaptation of the SADPP. She was also part of the project that aimed at screening school learners in Cape Town for NCDs. She is well- positioned and have needed experience for overseeing the success of the project. |
| Prof Andre Pascal Kengne | | South African Medical Research Council | Co-investigator: Prof Kengne is an Internist and Chronic diseases epidemiologist and preventionist. He is the current director of SAMRC’s NCD researcher unit and is the PI of the main SA-DPP. He has mentored both Dr. Yako and Dr. Hill and will provide the needed intellectual input to all aspects of the current project. |
| Dr. Simthandile Toni | | Mthatha General Hospital | Co-investigator/collaborator: Dr. Toni is a Medical practitioner at Mthatha General Hospital located at the O.R. Tambo district where the study will be conducted. He is an advocate of primary health care and disease prevention. This motivated him to develop a health promotion platform (SimTalk) that he is directing and hosting and aims at lowering the burden of diseases in the Eastern Cape province by opening a dialogue between health experts and community members.  His involvement in sport and experience as a Team Physician (under South African Football Association) will be valuable in designing appropriate physical exercises to be used in this intervention study.  Furthermore, being part of the proposed study will be beneficial in the implementation and sustainability of the SADPP to the entire OR Tambo district community, if found to be effective in decreasing the risk of T2D. |
| Prof. Constance Rusike | Walter Sisulu University | | Co-investigator: Dr. Rusike is an Academic researcher at Walter Sisulu University |
| Dr. Hannibal Musarurwa | Walter Sisulu University | | Co-investigator: Dr HT Musarurwa is an Academic researcher currently leading studies in HIV, Metabolic syndrome and the microbiome at Walter Sisulu University. He has active collaborations Austria, South Africa and the UK. He also has experience in research ethics, experimental design and data analysis and chemometrics. In this study he will actively participate in the design, assist in day-to-day activities of the project, and data analysis (as a Biostatistician). |

## **1.2 National and International Collaboration**

| **Name and Surname** | **Institution** | **Nature of collaboration** |
| --- | --- | --- |
| Prof Brian Oldenburg | University of Melbourne: Professor of NCD Control & Director of Centre for Health Equity, Melbourne School of Population and Global Health. | Co-investigator SA-DPP:  He is an international expert in NCD prevention and control in resource- constrained settings and is a PI on multiple projects in Africa and Asia, including the ongoing Indian Kerala Diabetes Prevention Project and a Global Alliance for Chronic Diseases (GACD) funded project evaluating the improved management and control of hypertension across three rural communities in India. He has extensive experience in multicentre community-based implementation studies (including CRT). He has collaborated with Profs Absetz and Valve on diabetes implementation in Europe and Asia for more than 10 years, and with Prof Kengne over the past five years in developing & establishing SA- DPP. Prof Oldenburg works closely with the PI and SA-DPP PROJECT TEAM to assist with the successful design and implementation of SA- DPP. He has visited South Africa regularly for research and other collaboration over the past 10 years. |
| Prof. Tandi Matsha | Cape Peninsula University of Technology: Head of Department & Chair Cardiometabolic Health | Collaborator on SA-DPP:  She has extensive experience doing research on cardio- metabolic diseases in Coloured communities in Cape Town. She is an ongoing mentor of Dr Yako |
| Prof Rajiv Erasmus | University of Stellenbosch: Head of the Division of Chemical Pathology | Collaborator on SA-DPP:  He has extensive experience on research relating to diabetes and cardio-metabolic diseases across Africa and has closely collaborated with Prof Matsha and Kengne in quantifying the cardio-metabolic risk of Coloured populations in Cape Town in recent years. He is a former mentor of Dr Yako |
| Prof. Naomi Levitt | University of Cape Town: Head of the Endocrinology and Diabetes Division | Collaborator on SA-DPP:  She has an impressive track record of primary care-based research on diabetes and NCDs, and of successful engagement with stakeholders on issues relating to NCDs. With Prof Puoane she is assisting the development and implementation of the project in Black townships. Her experience in conducting pragmatic CRT in those townships and in implementing text-messaging based intervention will be an advantage. |
| Prof. Thandi Puoane | University of the Western Cape: Emeritus Professor | Collaborator on SA-DPP:  She has extensively collaborated with Prof Kengne on diabetes in Africa, and with Prof Puoane and Dr Goedecke on NCD research in South Africa. She has an impressive track record of primary care-based research on diabetes and NCDs, and of successful engagement with stakeholders on issues relating to NCDs. |
| Prof. Julia Goedecke | South African Medical Research Council: Chief Specialist Scientist in the NCDRU | Co-investigator SA-DPP:  She has a very strong track-record on research relating to nutrition, physical activity, obesity and NCDs, particularly in Black South African populations. |
| Dr Nasheeta Peer | South African Medical Research Council: Specialist Scientist in the NCDRU | Co-investigator SA-DPP:  She has experience with population-based research studies having been involved with various diabetes and cardiovascular epidemiology projects in Cape Town. |
| Prof. Mieke Faber | South African Medical Research Council: Chief Specialist Scientist in the NCDRU | Co-investigator SA-DPP:  She has extensive experience in assessment of dietary intake, the food environment affecting dietary intake, dietary quality within the context of food and nutrition security, and food-based strategies for improved nutrition, particularly in infants and young children. She is assisting with the tailoring of dietary interventions requirements and tools for SA-DPP. |
| Ms. Esme Jordaan | South African Medical Research Council: Senior statistician at the Biostatistics unit | Consulting Statistician on SA-DPP |
| Dr Olufunke Alaba | University of Cape Town: Researcher/Lecturer at the Health Economist Unit, School of Public Health and Family Medicine | She is overseeing health economic analysis on the main SA-DPP, and will provide similar guidance on the study in the Eastern Cape |
